# Respiratory function and its predictive value for health related outcomes in the BELFRAIL cohort

**DOI:** 10.1186/2049-3258-73-S1-P11

**Published:** 2015-09-17

**Authors:** Eralda Turkeshi, Bert Vaes, Elena Andreeva, Catharina Mathei, Wim Adriaensen, Gijs Van Pottelbergh, Jean-Marie Degryse

**Affiliations:** 1 UCL, Brussels, Belgium; 2 IRSS, UCL, Brussels, Belgium; 3 KUL, Leuven, Belgium

## Background

Spirometry-based parameters of pulmonary function such as forced expiratory volume in 1 second (FEV1) have prognostic value beyond respiratory morbidity and mortality. Limited data are available on its prognostic value for adverse health outcomes in the growing group of very old adults (=80 years old). We investigate the prognostic value of FEV1 for adverse health outcomes in very old adults and assess the predictive value of airflow limitation (AL) for all-cause mortality and hospitalisation using two different approaches to cut-offs for FEV1/FVC (forced vital capacity).

## Methods

In a Belgian population-based, prospective cohort of 501 very old adults, survival, Cox and logistic regression multivariable analysis assessed the association of FEV1 standardizations with 5-year all-cause mortality, first hospitalization at 3 years and decline in mental and physical functioning at around 2 years. Survival and Cox regression analysis assessed the association of AL by the 5th percentile of GLI 2012 z-scores (GLI-LLN) and fixed (0.70) cut-offs with all-cause mortality and first hospitalisation.

## Results

Compared to the rest of the population, individuals in the lowest quartile of FEV1 standardizations had statistically significant increased adjusted risk for all-cause mortality (highest hazard ratio [HR] 1.96, 95% confidence interval [CI] 1.42-2.69) for FEV1/height cubed), first hospitalization (only FEV1/height cubed and height squared), decline in mental functioning (except FEV1 percent predicted). No FEV1 standardization was independently associated with physical decline. Only AL by GLI-LLN was independently associated with mortality (HR 2.10, 95% CI 1.30-3.38).

## Conclusions

In a cohort of very old adults, low FEV1 was found to be an independent predictor of all-cause mortality, hospitalization and decline in mental functioning. Only AL by GLI-LLN independently predicted all-cause mortality without missing individuals with significantly higher all-cause mortality and hospitalisation. Further research is needed on FEV1 as a potential risk marker for adverse health outcomes in very old adults.

